# A systematic review and meta-analysis of interventions to increase stroke thrombolysis

**DOI:** 10.1186/s12883-019-1298-2

**Published:** 2019-05-03

**Authors:** Mollie McDermott, Lesli E. Skolarus, James F. Burke

**Affiliations:** Neurology Department, University of Michigan, Cardiovascular Center, 1500 East Medical Center Drive – SPC #5855, Ann Arbor, MI 48109-5855 USA

**Keywords:** Acute ischemic stroke, Thrombolysis, IV tPA, Emergency medical services, Telemedicine, Public education

## Abstract

**Background:**

Although the efficacy of tissue plasminogen activator (tPA) for acute ischemic stroke is well established, rates of tPA use remain low. For clinicians, advocates, and policy-makers seeking to increase tPA treatment rates, it is important to understand what interventions exist and their relative effectiveness.

**Methods:**

We searched PubMed and EMBASE to identify all studies published between 1995 and January 8, 2015 documenting interventions to increase the use of tPA with broadly inclusive criteria. The principal summary measure was the percentage change in rate of tPA administration. Random effects meta-analytic models were built to summarize the effect of intervention compared to control overall and for intervention characteristics.

**Results:**

The search yielded 1457 results of which 25 met eligibility criteria. We identified 14 pre-post studies, ten randomized controlled trials, and one quasi-experiment. Included studies targeted their interventions at emergency medical services (EMS) (*n* = 14), telemedicine (*n* = 6), and public education (*n* = 6). In a random effects model, tPA administration was significantly higher in the intervention arm across all studies limiting enrollment to ischemic stroke patients (*n* = 16) with a risk ratio (RR) of 1.80 (95% confidence interval [CI], 1.45–2.22). A trend towards increased tPA administration was observed for all intervention approaches: risk ratio of 1.73 (95% CI, 1.44–2.09) for EMS, 1.58 (95% CI, 0.72–3.47) for telemedicine, and 1.89 (95% CI, 0.77–4.65) for public education, the latter not restricted to ischemic stroke patients.

**Conclusions:**

Interventions to increase tPA use appear to have considerable effectiveness. Our findings support the use of such interventions to improve stroke outcomes.

## Background

Although the efficacy of tissue plasminogen activator (tPA) for acute ischemic stroke is well established [1, 2], rates of tPA use remain low both nationally and globally. Estimates of tPA treatment rates in the United States are consistently less than 5% [3, 4]. ,and similar rates are found in the United Kingdom [5]. Given that stroke thrombolysis is either a cost-saving or highly cost-effective therapy, interventions to increase thrombolysis are likely to be sound societal investments [6–9].

Regional rates of tPA use range from 0 to 9.3%, suggesting both that regional factors may influence treatment rates and that considerable opportunity for improvement exists [10]. While the factors that underlie this variation are not well established, access to stroke care explains only a fraction of these differences [11]. Other factors potentially implicated in low treatment rates include poor symptom recognition by the public [12, 13]; failure of timely transport to emergency rooms [14, 15]; and underdeveloped emergency department (ED) systems of care [16, 17].

Interventions aimed at increasing tPA use have been developed to address these factors. These interventions use a variety of approaches in various settings to increase treatment rates. For clinicians, advocates, and policy-makers seeking to increase tPA treatment rates, it is important to both understand what interventions exist and to understand the relative effectiveness of these interventions. Addressing this knowledge gap may help hospitals and communities increase their acute stroke treatment rates.

To inform future initiatives to increase tPA use, we performed a systematic review and meta-analysis of existing interventions to increase tPA rates for acute ischemic stroke. Our primary goals were to: 1) quantify and describe intervention approaches and settings, 2) determine the overall effectiveness of these interventions, and 3) explore the relative effectiveness of particular intervention approaches.

## Methods

### Search strategy and selection criteria

We performed this study in accordance with the Preferred Reporting Items for Systematic Reviews and Meta-Analyses (PRISMA) statement [18]. First, we worked with an experienced research librarian to develop a pre-specified comprehensive search strategy for studies combining three major themes: stroke, tPA, and interventions to improve the rate of tPA use (search strings are listed in the Appendix). To capture as broad a sample of interventions as possible, we included all published studies regardless of the intervention target, intervention approach, intervention setting, and study design (with the exception of case reports). PubMED and EMBASE were separately searched. Only published studies were reviewed. All studies available in English and published between 1995 and January 8, 2015 were included.

**Fig. 1:**
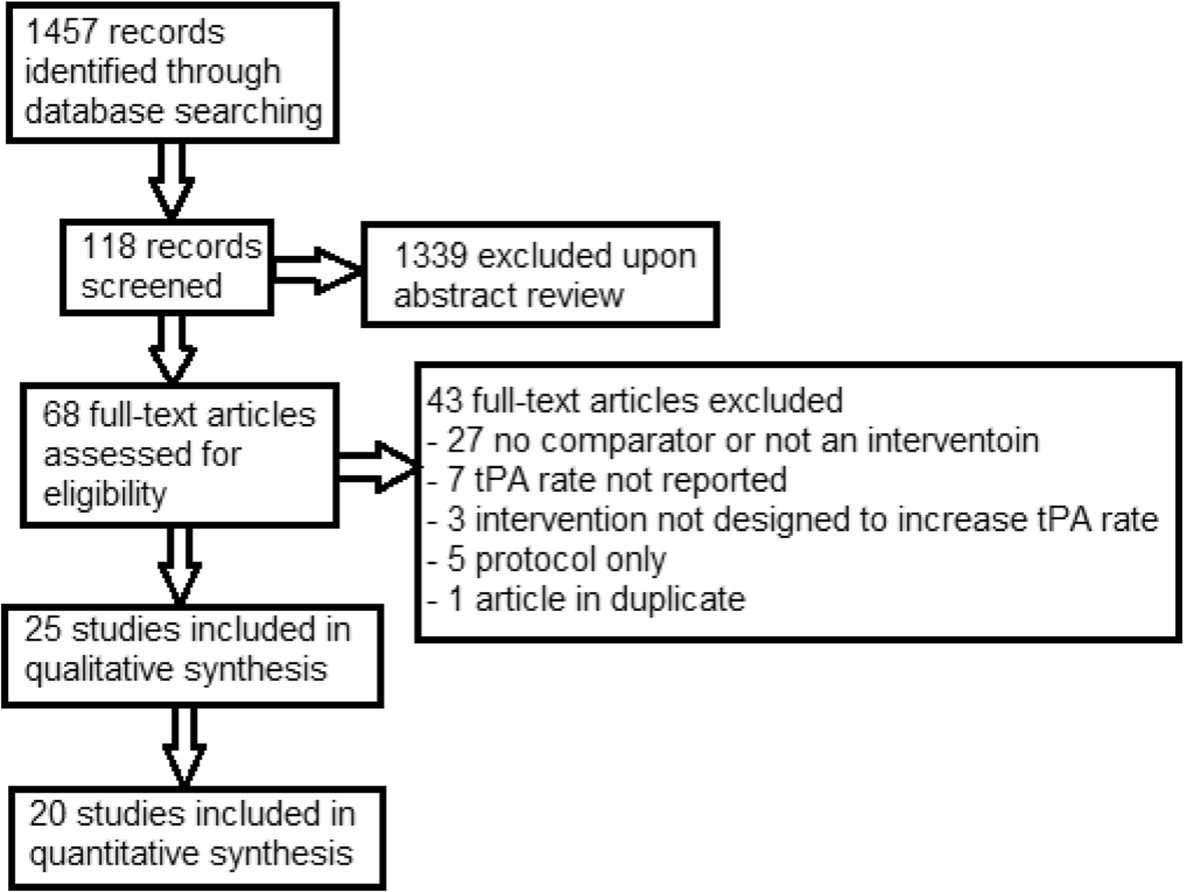
Flow chart of the article review process

After performing our search, we performed a staged abstract review as summarized in Fig. 1. Inclusion criteria were intentionally broad: 1) interventions involved acute stroke patients compared to a non-intervention comparator, and 2) interventions were, at least in part, intended to increase thrombolysis rates. An abstract was rejected only if it clearly failed to satisfy inclusion criteria.

All three authors (MM, LS, JB) reviewed the first 100 search results to identify candidate abstracts that would potentially meet the inclusion criteria. Agreement between reviewers was 100%. Subsequently, one author (MM) reviewed the remainder of the candidate abstracts. Next, MM reviewed the full text of each non-rejected article to arrive at final inclusion determinations. JB and LS each concurrently reviewed half of the non-rejected articles and compared their determinations to those made by MM. Overall inter-observer agreement was very good, kappa 0.87 (95% confidence interval [CI], 0.68–1.00).

### Data extraction

The following data were extracted from each study: study design, intervention approach (involvement of emergency medical services [EMS], telemedicine, public education, or other), intervention site (academic, community, or both), intervention setting (rural, urban, or both), intervention target (pre-hospital, intra-hospital, community, or other) comparison effect, intervention effect, number of patients in comparison, number of patients in intervention, and number of participating centers. Data were extracted by MM. The principal summary measure was the percentage change in rate of tPA administration attributed to the intervention.

### Quality assessment

The three authors analyzed each included study using the GRACE checklist [19]. The GRACE checklist consists of 11 yes/no questions that address the key components of observational studies of comparative effectiveness. This process was performed iteratively. First each author reviewed ten studies and then the group met for a consensus conference. After the consensus conference, LS and MM reviewed each of the remaining studies. Disagreements were resolved by group consensus.

### Statistical analysis

After completing the systematic review, we performed a meta-analysis. For studies of related or overlapping study populations, we prioritized studies that were not pilots, had the largest sample size, and/or were the first publication for an intervention. Summary effects were estimated by building fixed and random effects meta-analytic models to estimate summary risk ratios of the effect of intervention compared to control. We evaluated summary effects both in the population of all included studies as well as in the subset of studies with results available for ischemic stroke patients (rather than both ischemic and hemorrhagic stroke patients, the latter of whom clearly have a contraindication to intravenous tPA). Heterogeneity was summarized by calculating I^2^ statistics, which describes the degree of variability between studies that is due to heterogeneity as opposed to random error. Given our broad inclusion criteria, we anticipated that significant heterogeneity would exist in intervention approaches and settings. Consequently, we followed the Cochrane handbook for systematic reviews of interventions and considered the random effects model as our primary analysis. Funnel plots were generated to explore potential publication bias. Analyses were performed in R version 3.1.0 and meta-analysis performed with the ‘meta’ package 4.1–0 [20].

Studies were grouped based on pre-specified intervention characteristics including setting (rural versus urban), site (community versus academic), and intervention approach – utilization of telemedicine (yes versus no), incorporation of public education (yes versus no), and involvement of EMS (yes versus no). Similar models were repeated for the subset of studies with each of our pre-specified study characteristics. We found that one study, the Target:Stroke initiative, had by far the largest sample size of any included study (over one million subjects) and thus had a disproportionately large influence on the summary effects [21]. We thus performed post-hoc analyses to explore the extent to which the Target:Stroke initiative influenced summary effects by repeating our primary analysis excluding Target:Stroke.

## Results

### Summary of included studies

**Fig. 2:**
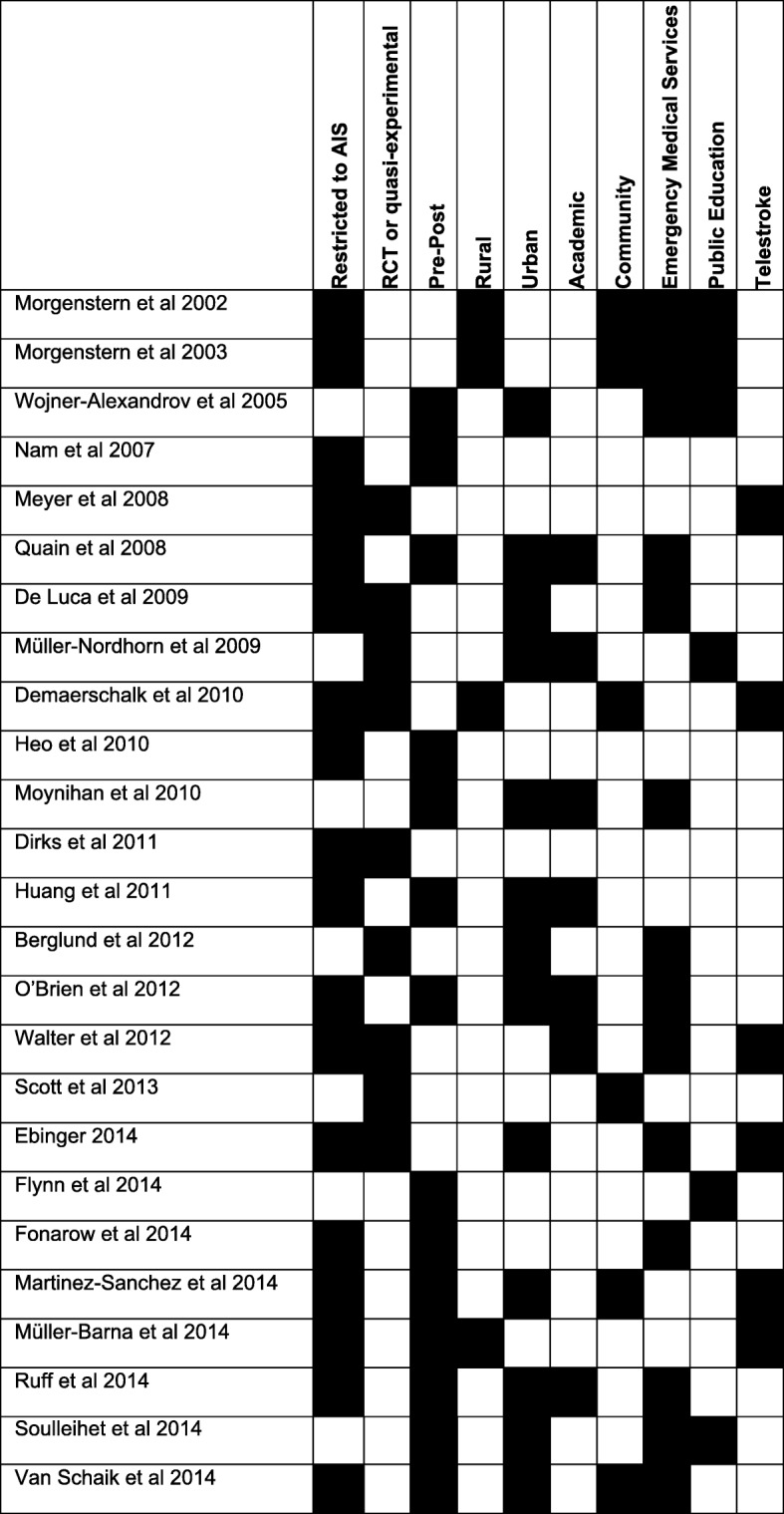
Characteristics of the 25 studies meeting inclusion criteria for our meta-analysis

Our search of PubMed and EMBASE yielded 1457 results of which 68 were deemed appropriate for full-text review (Fig. 1). Of these, 25 met the inclusion criteria (Fig. 2, Table 1). We identified 14 pre-post studies, ten randomized controlled trials (RCTs), and one quasi-experiment. A total of 18 studies reported results of ischemic (rather than ischemic and hemorrhagic) stroke patients. Studies utilized a variety of different intervention approaches including involvement of EMS (*n* = 14), telemedicine (*n* = 6), and public education (*n* = 6) (Table 2). Intervention settings (when included, *n* = 22) were urban (*n* = 13), rural (*n* = 4), and combined (*n* = 5). Study centers (when included, *n* = 19) were academic (*n* = 7), community (*n* = 6), and combined (*n* = 6). Although it was not a pre-specified subgroup analysis, we found a number of studies that investigated a hub-and-spoke acute stroke care model (*n* = 4), typically with co-utilization of telemedicine (*n* = 3).

**Table 1:** Characteristics of the 25 included studies (unc = unclear; RCT = randomized controlled trial)

	Study Location	Design	Controls	Primary Intervention	# in control / # in intervention	IV tPA rate in intervention (%)	IV tPA rate in control (%)
Morgenstern et al 2002	rural	quasi-experimental	concurrent	multilevel stroke education	233/266	8.65	0.9
Morgenstern et al 2003	rural	quasi-experimental	concurrent	multilevel stroke education	70/80	11.2	1.4
Wojner-Alexandrov et al 2005	urban	pre-post	historical	multilevel stroke education	198/533	12	10.6
Nam et al 2007	unc	pre-post	historical	computerized physician order entry	529/213	11.7	2.6
Meyer et al 2008	Rural and urban	RCT	concurrent	video consultation	103/102	24	30
Quain et al 2008	urban	pre-post	historical	pre-hospital acute stroke triage protocol	107/140	21.4	4.7
De Luca et al 2009	urban	cluster-RCT	concurrent	pre-hospital acute stroke triage protocol	115/175	8.6	1.7
Müller-Nordhorn et al 2009	urban	cluster-RCT	concurrent	direct-to-community member educational material about stroke symptoms and calling EMS	741/647	2.9	2.3
Demaerschalk et al 2010	rural	RCT	concurrent	telestroke	27/26	31	30
Heo et al 2010	urban	pre-post	historical	computerized physician order entry	5798/5405	5.8	3.4
Moynihan et al 2010	urban	pre-post	historical	Introduction of a hub and spoke model	unc/unc	6	1.2
Dirks et al 2011	rural and urban	cluster-RCT	concurrent	meetings at trial hospitals based on the Breakthrough Series model	2140/2483	16	14
Huang et al 2011	urban	pre-post	historical	less restrictive exclusion tPA treatment criteria	333/128	6.25	5.1
Berglund et al 2012	urban	RCT	concurrent	increased pre-hospital priority level for patients with stroke	454/488	12.3	5.3
O’Brien et al 2012	urban	pre-post	historical	pre-hospital acute stroke triage protocol	67/115	19.1	7.5
Walter et al 2012	rural and urban	RCT (randomized week)	concurrent	mobile stroke unit	40/49	25	20
Scott et al 2013	rural and urban	cluster-RCT	concurrent	hospital-based stroke education	9222/8419	2.8	2.1
Ebinger 2014	urban	RCT (randomized week)	concurrent	mobile stroke unit	1041/1070	29	21.1
Flynn et al 2014	unc	pre-post	historical	mass media stroke awareness campaign	Unc/unc	unc	unc
Fonarow et al 2014	rural and urban	pre-post	historical	Target:Stroke initiative	541,358/479,281	8.1	5.7
Martinez-Sanchez et al 2014	urban	pre-post	historical	telestroke	259/225	8	4.7
Müller-Barna et al 2014	rural	pre-post	historical	telestroke	2466/4409	15.5	2.6
Ruff et al 2014	urban	pre-post	historical	implementation of 10 best practices in single center	1413/925	15.4	8.2
Soulleihet et al 2014	urban	time series analysis	none	public awareness campaigns	unc/unc	unc	unc
Van Schaik et al 2014	urban	pre-post	historical	pre- and intra-hospital acute stroke triage protocol	828/917	9.8	4.95

*unc* unclear; *RCT* randomized controlled trial

**Table 2:** Included studies by intervention approach

Intervention	Studies Utilizing Intervention
Involvement of Emergency Medical Services (*n* = 14)	Morgenstern 2002; Morgenstern 2003; Wojner-Alexandrov 2005; Quain 2008; De Luca 2009; Moynihan 2010; Berglund 2012; O’Brien 2012; Walter 2012; Ebinger 2014; Fonarow 2014; Ruff 2014; Soulleihet 2014; Van Schaik 2014
Telestroke (*n* = 6)	Meyer 2008; Demaerschalk 2010; Walter 2012; Ebinger 2014; Martinez-Sanchez 2014; Muller-Barna 2014
Public Education (*n* = 6)	Morgenstern 2002; Morgenstern 2003; Wojner-Alexandrov 2005; Muller-Nordhorn 2009; Flynn 2014; Soulleihet 2014

### Summary of study quality

Of the 11 GRACE criteria, five (was treatment exposure adequately recorded, was the primary outcome objectively measured, was the study restricted to new initiators of treatment, were comparisons concurrent or were historical comparisons justified, and was the classification of exposed and unexposed subjects free of immortal time bias) were met by all 25 studies. Fifteen studies satisfied the criterion that important covariates that may be confounders be available and recorded; nine that these confounders be taken into account in the study design or analysis. For example, Dirks et al. reported an effect size cluster-adjusted for center characteristics including hospital size, academic versus nonacademic, and previous thrombolysis rate at hospital level; as well as for patient characteristics such as age, sex, stroke severity, and comorbidities [22]. Nine studies satisfied the criterion that the primary outcome be measured or identified in an equivalent manner between groups. Only six studies met the criterion that meaningful analyses be performed to test the key assumptions on which the primary results were based. For example, Scott et al. examined whether there were differences between the intervention and control hospitals in the pre- versus post-intervention phases in neurologists and neurosurgeons on staff, primary stroke center status, and residency training site status [23].

### Meta-analysis results

**Fig. 3:**
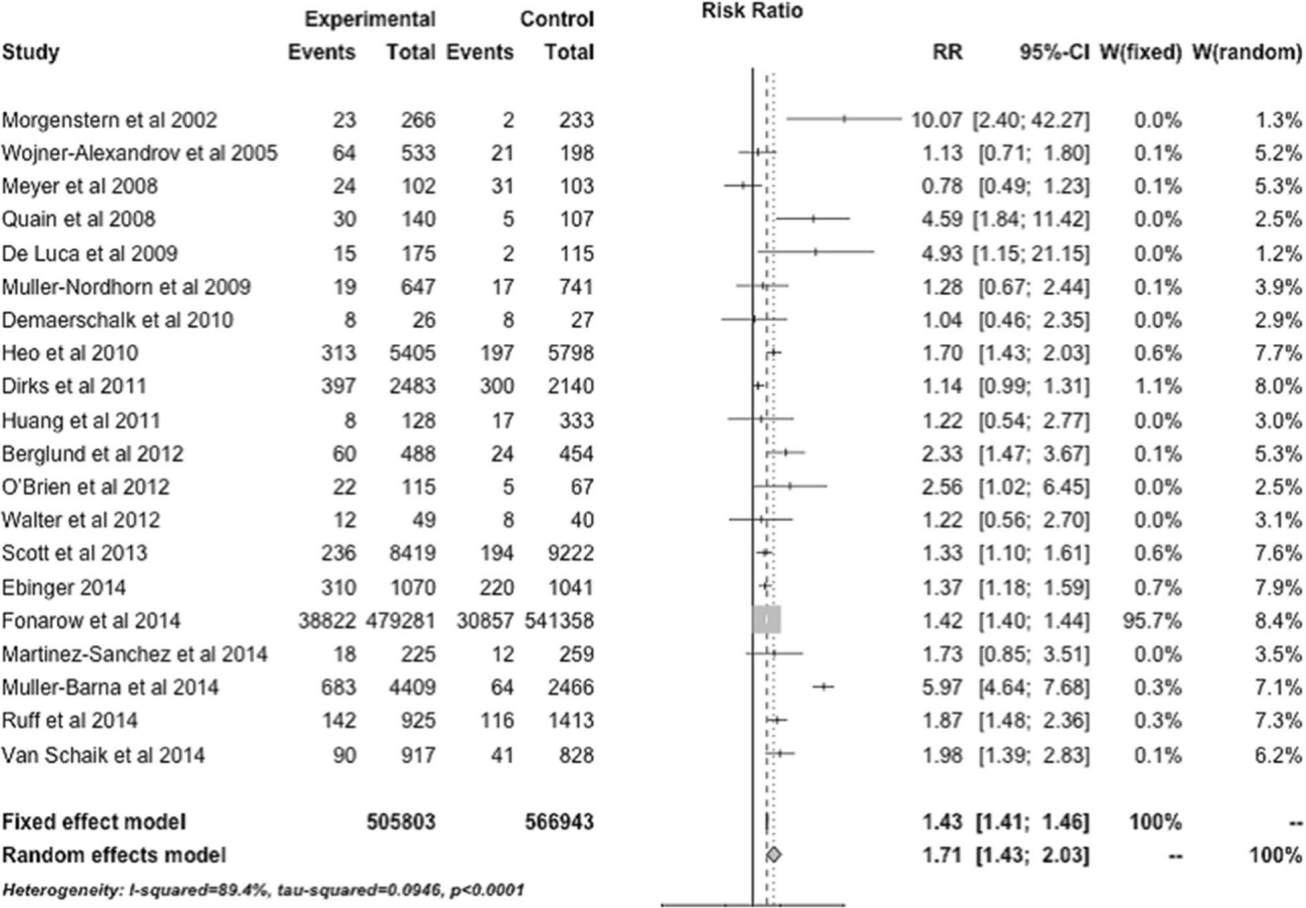
Treatment effects of all interventions to increase tissue plasminogen activator (tPA) utilization across all 25 included studies

**Fig. 4:**
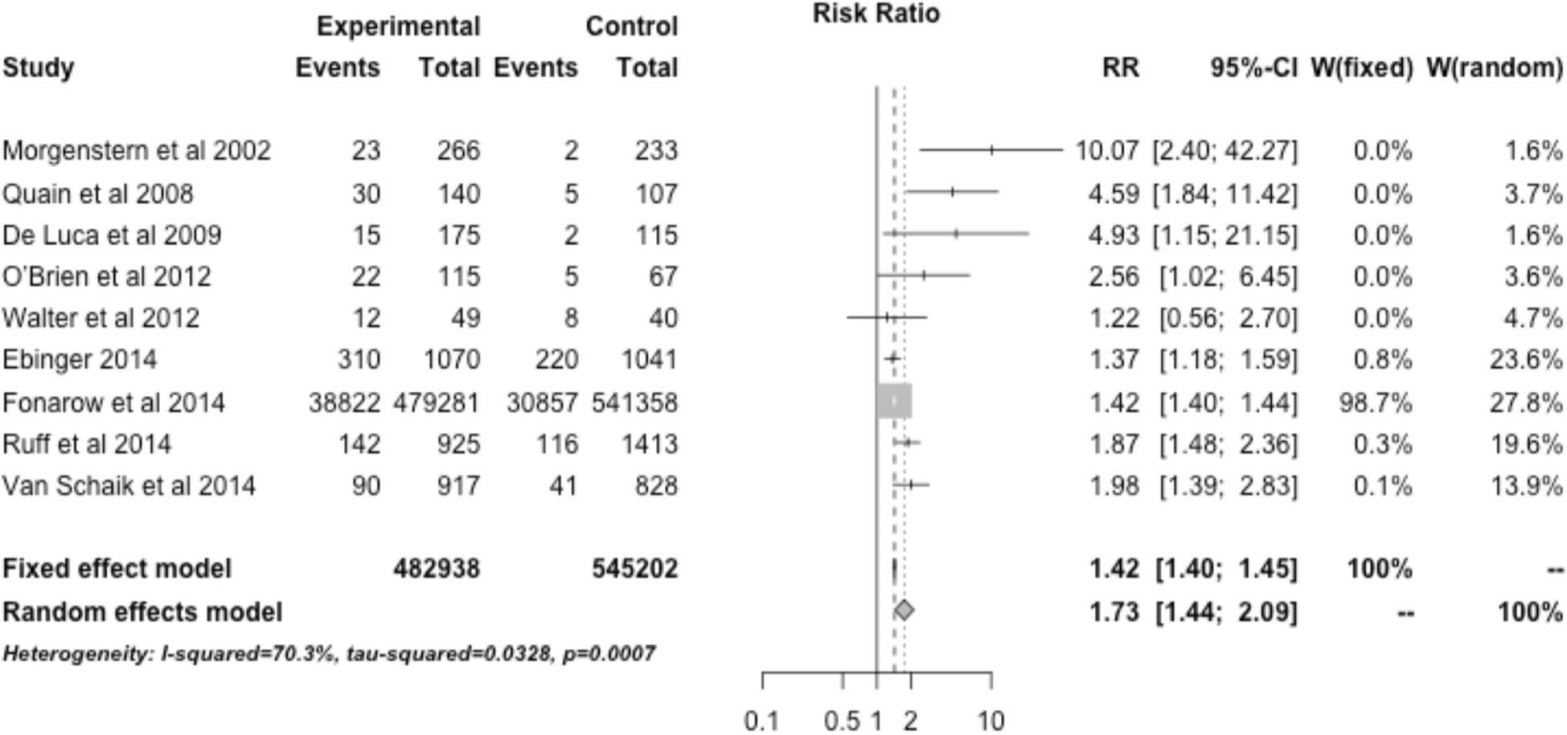
Treatment effect of interventions restricted to ischemic stroke patients that specifically involved emergency medical services (EMS) across 9 relevant studies

In the random effect model, thrombolysis use was significantly higher in the intervention arm across all 25 studies (Fig. 3, RR [risk ratio] = 1.71; 95% CI, 1.43–2.03) and across all studies restricted to ischemic stroke patients (RR = 1.80; 95% CI, 1.45–2.22) compared to controls. Pre-post studies showed a RR of 2.19 (95% CI, 1.54–3.11) and RCTs a RR of 2.00 (95% CI, 1.36–2.94) (Fig. 4).

**Fig. 5:**
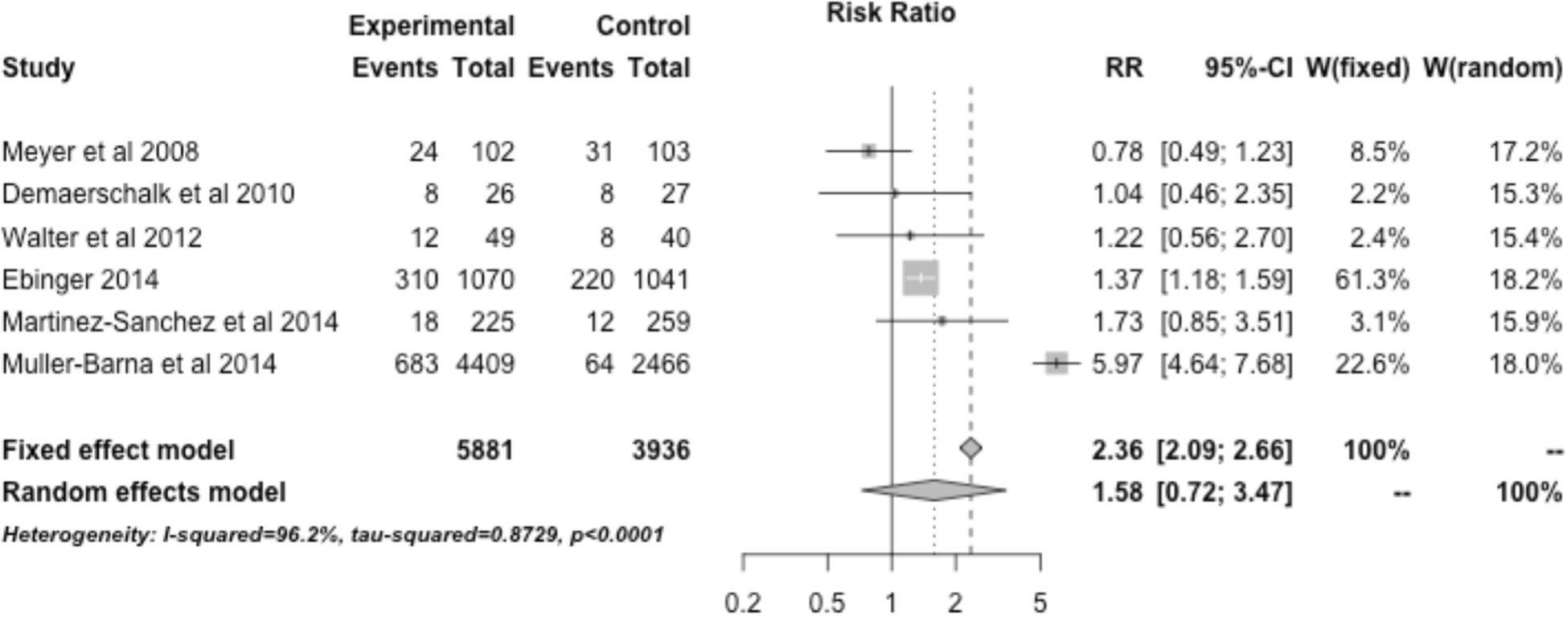
Treatment effect of interventions restricted to ischemic stroke patients that specifically involved telestroke across 6 relevant studies

**Fig. 6:**
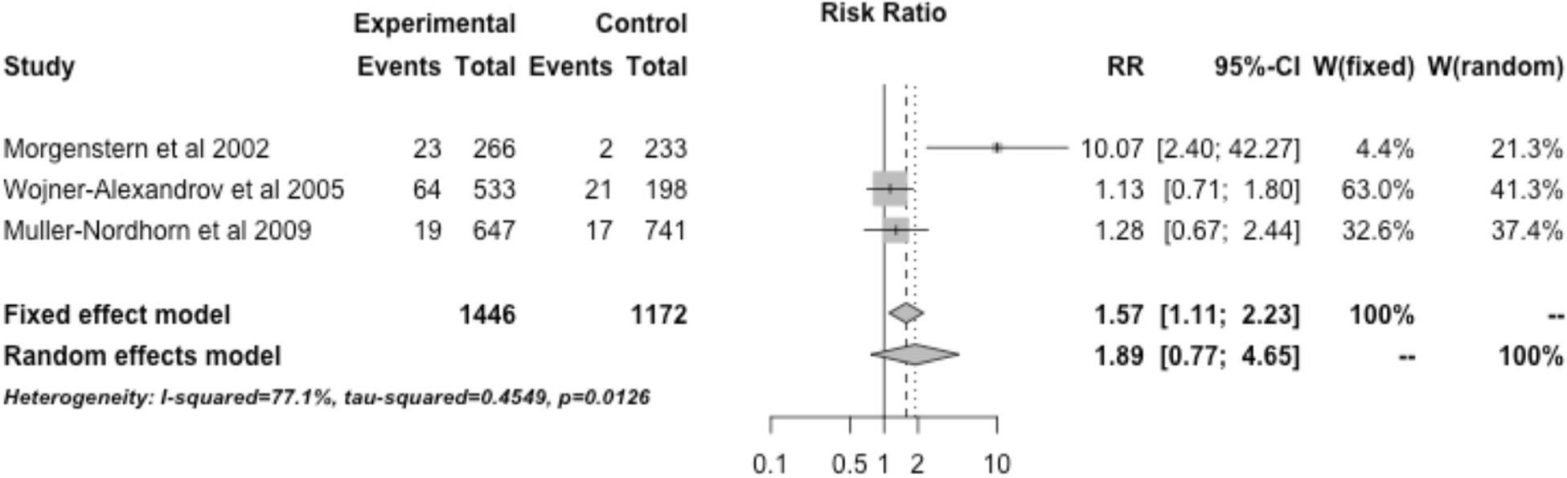
Treatment effect of interventions restricted to ischemic stroke patients that specifically involved public education across 3 relevant studies

Interventions restricted to ischemic stroke patients that specifically involved EMS were associated with an increased rate of tPA administration with a RR of 1.73 (95% CI, 1.44–2.09). Interventions restricted to ischemic stroke patients that involved telemedicine were associated with an increased rate of tPA administration with a RR of 1.58 (95% CI, 0.72–3.47) (Fig. 5). The three studies utilizing public education (not restricted to ischemic stroke) showed a RR of 1.89 (95% CI, 0.77–4.65) (Fig. 6).

As mentioned above, we repeated our primary analysis with exclusion of Target:Stroke. Across all 24 remaining studies, thrombolysis use remained significantly higher in the intervention arm with a RR of 1.77 (95% CI, 1.38–2.27). Across studies restricted to ischemic stroke patients, the RR increased slightly to 1.90 (95% CI, 1.39–2.59). Studies involving EMS also trended toward an increasing RR of 2.91 (95% CI, 1.61–5.26) after exclusion of Target:Stroke.

**Fig. 7:**
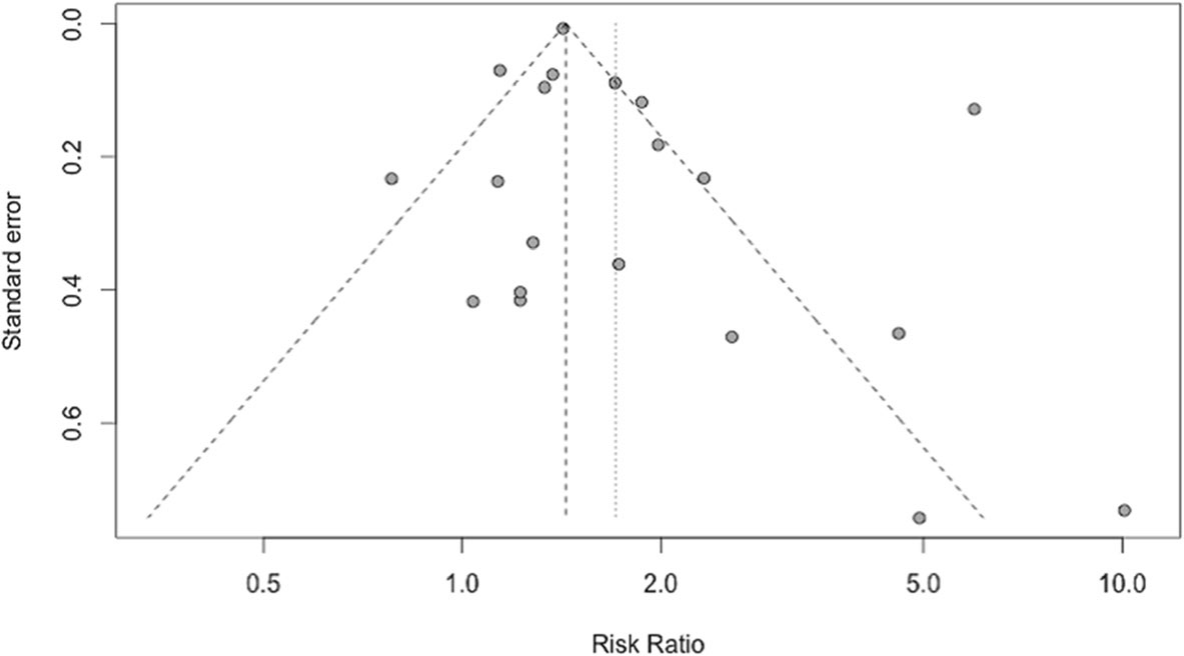
Funnel plot of effect size by standard error (surrogate for study size) across all studies

A funnel plot of all studies (Fig. 7) explored the potential for publication bias in our sample. The studies with the largest standard error (standard error > 0.45) reported four of the five largest effect sizes. Medium-sized studies (standard error 0.45 to > 0.15) showed a similar discrepancy. Qualitative assessment of the funnel plots reveals potential asymmetry in the reporting of large standard error studies – the four studies with the highest standard error all reported larger than average effect sizes. This suggests possible underreporting of small negative studies or studies with small effect sizes.

## Discussion

In this systematic review, we found numerous interventions that used a variety of approaches to increase tPA treatment rates in different settings. The intervention studies were reasonably designed to address the effectiveness of the tested interventions. The magnitude of benefit of the average intervention was substantial, and a trend toward benefit was observed across all intervention approaches studied. The overall summary risk ratio of 1.80 means that, if a region has a 5% treatment rate, this rate would increase to 9% with the average intervention. However, whether this effect size is an accurate representation of the real world effect of all attempted interventions is less clear.

Our review uniquely contributes to the literature by comprehensively exploring interventions that have been utilized to increase thrombolysis use. As such, our analysis attempts to inform the question for a region or hospital about how best to increase tPA treatment rates. We did not find strong evidence that one intervention approach was associated with substantial differences in treatment rates compared to other intervention approaches as the point estimates were similar across intervention type. Given the small number of included studies and their heterogeneity, the data do not support strong conclusions about which intervention approach is best. Because interventions involving EMS were most common, the statistical evidence is most robust for EMS-based interventions. Therefore, of all the interventions identified, it is most likely that EMS-based interventions are efficacious. However, we cannot assume that the benefit of EMS-based interventions is of greater magnitude than the benefit of other intervention approaches.

Rather than selecting an intervention approach, a region seeking to maximize improvement in its treatment rates may wish to first determine its largest treatment barriers and then accordingly select a strategy, as it appears that most intervention approaches have relatively similar overall effects. For regions with long pre-hospital delay – focus on public education; for regions with prolonged time from EMS activation to ED arrival – focus on EMS; for regions without access to adequate tPA support – focus on telemedicine, and for regions with long door to treatment times – focus on hospital quality improvement. As many of the interventions suggest, focusing on more than one barrier at a time is eminently feasible.

Once regional barriers are identified, a number of excellent exemplar interventions exist. In their Stockholm-based study, Berglund et al. found that simply increasing the EMS priority level of stroke patients from two to one resulted in a 14% absolute increase in treatment rates (*p* < 0.001) [24]. Quain et al. implemented a Pre-hospital Acute Stroke Triage (PAST) protocol, which included a pre-hospital stroke assessment tool for EMS, an established protocol for hospital bypass, and pre-hospital notification. The rate of thrombolysis in that study increased from 4.7% pre-intervention to 21.4% post-intervention (*p* < 0.001) [25]. In their Bavaria-based study, Müller-Barna et al. studied the effectiveness of the TeleMedical Project for integrative Stroke Care (TEMPiS), an effort which sought to improve stroke outcomes in rural areas. This initiative consisted of 24/7 virtual availability of a vascular neurologist at 15 regional hospitals [26]. Rates of tPA administration increased from 0.4% in the year before the intervention (2002) to 15.5% in the intervention’s tenth year (2012). Finally, the TLL Temple Foundation Stroke Project used a public education approach to improve acute stroke recognition and treatment by employing a community-based mass media campaign, community volunteer training, and the establishment of multidisciplinary teams in hospitals to develop ED protocols [27]. In this study, the percentage of ischemic stroke patients treated with IV tPA was 8.65% in the intervention group compared to 0.86% in the control group. In addition to the interventions identified in our review, since completion of the review, the results of additional studies have suggested that mobile stroke units are effective in increasing tPA treatment rates in selected regions [28–30].

Our study has several limitations. The pattern of missing data suggests publication bias which may result in an overestimate of the summary effect size. If all interventions were published, we would anticipate that small studies with relatively large positive effects would have similar representation to small studies with relatively small effects. However, we found that the smallest studies in our sample universally had large positive effects, suggesting publication bias. On the other hand, there was no evidence that effect sizes varied by study design, as similar effect sizes were seen in the strongest study designs (cluster RCTs) and the rest of the reported studies. In addition, the statistics for heterogeneity among studies is high given our analysis of varied interventions. Interpretation of the pooled effect sizes should be made with caution due to the heterogeneity among the studies. We have followed the Cochrane handbook for systematic reviews of interventions by reporting our random effects analysis as our primary analysis as it better accounts for heterogeneity.

Thirteen of the 25 studies in our review were pre-post design. Pre-post studies can be biased as data may be differently collected (e.g. more systematically) after an intervention than before an intervention.

Furthermore, many governmental, regional, and organizational interventions to improve access to thrombolysis are not published as studies or trials in journal articles. Our meta-analysis fails to capture these interventions. In addition, because our literature review was completed in 2015, our analysis includes limited studies about the effectiveness of mobile stroke units in increasing thrombolysis rates [31, 32].

Given the magnitude of the summary effect size, the number of studies included, and the consistency of the positive effect, it is likely that interventions have a net positive effect of a magnitude that is clinically and societally meaningful. We did not prospectively register our meta-analysis with a register given that, at the time we undertook this study, meta-analysis registration was not widely performed.

In summary, a considerable body of evidence exists supports a variety of interventions to increase tPA use. On the whole, these interventions appear to have considerable effectiveness and our findings support the pursuit of future interventions to increase the rate of tPA administration.

## Appendix

Search strings.

Stroke:

stroke [mh] OR stroke [tiab] OR strokes [tiab] OR (cerebrovascular disorders [mh] AND (stroke [tiab] OR strokes [tiab]) AND (1988 [pdat]: 2000 [pdat])).

tPA:

thrombolytic therapy [mh] OR thrombolysis [tw] OR “thrombolytic therapy” [tiab] OR tissue plasminogen activator [mh] OR “tissue plasminogen activator” [tw] OR tpa [tiab] OR ttpa [tiab] OR rtpa [tiab] OR r-tpa [tiab] OR t-tpa [tiab].

Study design or publication types:

randomized controlled trials as topic [mh] OR randomized controlled trial [ptyp] OR “randomized controlled” [tiab] OR “randomised controlled” [tiab] OR “cluster randomized” [tiab] OR “cluster randomised” [tiab] OR “cluster randomization” [tiab] OR “cluster randomisation” [tiab] OR “before and after study” [tiab] OR “before and after studies” [tiab] OR interrupted time series analysis [mh] OR “interrupted time series” [tiab] OR “pre-post” [tiab] OR “pre and post” [tiab] OR pre/post [tiab] OR preintervention [tiab] OR pre-intervention [tiab] OR “difference in differences” [tiab] OR quasiexperimental [tiab] OR quasi-experimental [tiab] OR “stepped wedge” [tiab] OR (uncontrolled [tiab] AND observational [tiab]) OR evaluation studies as topic [majr] OR evaluation studies [ptyp].
